# Measurement of Fecal Testosterone Metabolites in Mice: Replacement of Invasive Techniques

**DOI:** 10.3390/ani10010165

**Published:** 2020-01-18

**Authors:** Kerstin E. Auer, Marius Kußmaul, Erich Möstl, Katharina Hohlbaum, Thomas Rülicke, Rupert Palme

**Affiliations:** 1 Department for Biomedical Sciences, Institute of Laboratory Animal Science, University of Veterinary Medicine Vienna, Veterinärplatz 1, 1210 Vienna, Austria; 2 Department for Biomedical Sciences, Unit of Physiology, Pathophysiology and Experimental Endocrinology, University of Veterinary Medicine Vienna, Veterinärplatz 1, 1210 Vienna, Austria; 3 Department of Veterinary Medicine, Institute of Animal Welfare, Animal Behavior and Laboratory Animal Science, Freie Universität Berlin, Königsweg 67, 14163 Berlin, Germany

**Keywords:** testosterone, mice, androgens, non-invasive, feces

## Abstract

**Simple Summary:**

Testosterone is the main reproductive hormone in male vertebrates. Conventional methods to assess testosterone rely on invasive blood sampling procedures, which can induce pain and distress to the animals. Here we successfully validated a non-invasive method to determine testosterone levels by assessing testosterone metabolites (TMs) in excreta of mice. We investigated the effects of sex and daytime on the metabolism and the excretion of TMs and validated the applied EIA to measure TMs. Further, we assessed diurnal fluctuations in TM excretions in both sexes and across strains. We found that males excreted more radiolabeled TMs via their feces compared to females. TM excretion patterns did not differ between sexes but TM excretion occurred faster in urinary than fecal samples. Animals excreted TM faster during the night than during the day. Daytime had no effect on the formed TMs; however, males and females formed different TMs. As expected, males showed higher fecal TM levels than females. Males also showed diurnal fluctuations in their TM levels but we found no differences between mouse strains. Our non-invasive method to assess fecal TMs can be widely used and will benefit various research disciplines.

**Abstract:**

Testosterone is the main reproductive hormone in male vertebrates and conventional methods to measure testosterone rely on invasive blood sampling procedures. Here, we aimed to establish a non-invasive alternative by assessing testosterone metabolites (TMs) in fecal and urinary samples in mice. We performed a radiometabolism study to determine the effects of daytime and sex on the metabolism and excretion pattern of radiolabeled TMs. We performed physiological and biological validations of the applied EIA to measure TMs and assessed diurnal fluctuations in TM excretions in male and female mice and across strains. We found that males excreted significantly more radiolabeled TMs via the feces (59%) compared to females (49.5%). TM excretion patterns differed significantly between urinary and fecal samples and were affected by the daytime of ³H-testosterone injection. Overall, TM excretion occurred faster in urinary than fecal samples. Peak excretion of fecal TMs occurred after 8 h when animals received the ^3^H-testosterone in the morning, or after 4 h when they received the ^3^H-testosterone injection in the evening. Daytime had no effect on the formed TMs; however, males and females formed different types of TMs. As expected, males showed higher fecal TM levels than females. Males also showed diurnal fluctuations in their TM levels but we found no differences in the TM levels of C57BL/6J and B6D2F1 hybrid males. Finally, we successfully validated our applied EIA (measuring 17β-hydroxyandrostane) by showing that hCG (human chorionic gonadotropin) administration increased TM levels, whereas castration reduced them. In conclusion, our EIA proved suitable for measuring fecal TMs in mice. Our non-invasive method to assess fecal TMs can be widely used in various research disciplines like animal behavior, reproduction, animal welfare, ecology, conservation, and biomedicine.

## 1. Introduction

Testosterone is one of the major sex hormones produced by the body, occurring in both males and females. In males, testosterone promotes territorial aggression, courtship, and sexual behavior, as well as sperm and secondary sexual trait production [1]. Also, testosterone has been shown to directly affect other fitness parameters such as immune function [1]. In females, the role of testosterone is less well-investigated, though effects on female health [2], sexual behavior [3,4], and aggression have been reported in various species [5,6,7], highlighting the overall significance of testosterone in behavior, reproduction, and health.

Conventional methods to measure testosterone and other hormones rely on invasive blood sampling procedures. Besides raising ethical concerns, blood sampling can be problematic—especially for small animals like mice and rats—as the invasive sampling procedure itself can alter hormone secretion, only small volumes can be collected and repeated sampling is not advisable or sometimes even impossible [8,9]. Moreover, blood sampled hormone concentrations represent a snapshot of the hormone status and whenever hormone levels show strong episodic or diurnal fluctuations such results will be misleading. A non-invasive alternative that circumvents all these limitations is the determination of hormone metabolites in excreta. Collecting feces and urine is easy and can be repeatedly performed without disturbing the animal’s behavior or interfering with its endocrinological status. Also, fecal and urinary samples are less affected by episodic fluctuations in hormone secretions and thus might reflect hormone levels more robustly [10,11,12,13].

Even though there are several advantages for using excreta to assess hormone levels, this method is not straight-forward and needs careful establishment. Firstly, as the circulating hormone is heavily metabolized prior to excretion, it is essential to find and validate a group-specific enzyme immunoassay (EIA) for the quantification of these metabolites [14,15]. Immunoassays designed for blood samples target actual hormones rather than their metabolites and are thus less suited for analyzing fecal or urinary samples. Secondly, when measuring hormone metabolites in excreta one has to consider the required intestinal gut passage time, i.e., time delay between hormone secretion and peak excretion of its metabolites, to correctly interpret results [13]. The intestinal gut passage time is species specific and can vary depending on sex, age, activity pattern and even diet (for review see [12,13,15]). Also, hormone metabolites can be excreted via feces or urine and differences in the route of excretion have been reported between species and sexes [13,16]. Taken together, because metabolism and excretion of hormones differ significantly between species and sometimes even between sexes, these non-invasive methods for measuring hormone metabolites in excreta have to be validated for each species before application [10,12,13,15,17].

In the last years, the assessment of fecal steroid hormone metabolites has been established in an increasing number of species although most studies focus on glucocorticoids (for review see [12]) rather than sex steroids. So far, the assessment of fecal testosterone metabolites (TMs) has been successfully validated in male and female red squirrels (*Tamiasciurus hudsonicus*) [18,19], chinchilla (*Chinchilla sp.*) [20], bank voles (*Myodes glareolus*) [21], baboons (*Papio cynocephalus*) [22], and three other species of nonhuman primates [23]. Few studies have used fecal and urinary samples for TM measurement in mice and to our knowledge a proper physiological and biological validation of the method to assess TMs in this species is still lacking. Billitti et al. [24] took a first approach in performing a radiometabolism study; however, the small number of animals used (n = 3) and the lack of information regarding the experimental mice and the sampling procedure (e.g., the authors did not state which mouse strain was used or at what day time feces were collected) limits the applicability of their findings. Another working group adapted an EIA used to target plasma steroid hormones including testosterone for fecal and urinary samples [25]. The authors did not attempt to identify respective steroid hormone metabolites and did not perform any validation for their assay besides providing standard curves. In subsequent studies the authors then used their assay for urinary rather than fecal samples [26,27,28], which seems problematic as steroid conjugates prevail in urinary samples and are more complicated to quantify.

Here we performed a comprehensive study where we investigated testosterone metabolism in mice and established and validated an enzyme immunoassay to reliably assess individual testosterone levels. We injected radiolabeled testosterone to assess the effects of sex and time of day on the route and excretion pattern of TMs. There is only little information available about sex-specific and diurnal effects on patterns of TM excretion in any species even though such factors can be relevant for studies investigating hormone metabolites in excreta in various species, not only in mice. We further characterized the radiolabeled metabolites in feces with a reversed-phase HPLC to validate our assay. As an additional validation approach, we performed a pharmacological stimulation with human chorionic gonadotropin (hCG) and performed castrations to verify that the TMs detected with our EIA follow the respective manipulations [14,17]. Finally, we also determined normal diurnal fluctuations in fecal TMs in males and females and collected feces from two different isogenic mouse strains to determine whether strain specific variation in excretion patterns exists. Mice are the most commonly-used model organism in biomedical research and our study provides a non-invasive alternative to assess individual testosterone levels and extends the area of application of mice as a model organism. Also, a validated method for fecal TM measurement will be useful for other fields of research like reproductive physiology and behavioral and wildlife ecology.

## 2. Materials and Methods

### 2.1. Experimental Animals

#### 2.1.1. Radiometabolism Study, Diurnal Fluctuations and hCG Challenge

In total, we used 32 C57BL/6JRj (*Mus musculus f. domesticus*, 16 males, 16 females) and 8 male B6D2(C57BL/6JRj × DBA/2JRj)F1 hybrid mice which were purchased from Janvier Laboratories (Staint-Berthevin Cedex, France) at the age of 8 weeks. Sample sizes were estimated with power statistics based on similar studies assessing fecal corticosterone metabolites in mice [16,29]. Upon arrival, animals from the same strain were housed in same sex groups in mouse open top cages (type IIL, 36.5 × 20.7 × 14 cm, Tecniplast, Buguggiate, Italy). Cages were equipped with wooden bedding (LIGNOCEL^®^ 3–4 S, J. Rettenmaier and Söhne GmbH + Co. KG, Rosenberg, Germany), nesting material (Pur-Zellin 4 × 5 cm; Paul Hartmann GmbH, Wiener Neudorf, Austria) and cardboard tubes (7.6 × 3.8 cm diameter, Special Diet Service, Claus GmbH, Limburgerhof, Germany). Commercial mouse diet (ssniff^®^, V1534, Soest, Germany) and tab water were provided *ad libitum*. Ten days prior to the start of the experimental sample collections, mice were housed individually to control fluctuations in testosterone levels related to social interactions and to habituate mice to the single housing condition during the experiments. Standard laboratory conditions (temperature 21 ± 1 °C, humidity 40–55%, light-dark cycle: 12:12 h, lights on at 8:00 a.m.) were maintained in the colony and during the experiment.

#### 2.1.2. Castration Experiment

In total 19 male C57BL/6JRj mice which were purchased from Janvier Laboratories (Staint-Berthevin Cedex, France) were used at the age of 18 to 42 weeks. These mice had previously been used in another experiment [30] and had been individually housed for 6 to 32 weeks in mouse type III open top cages (42.5 × 27.6 × 15.3 cm, Tecniplast, Buguggiate, Italy). Cages were equipped with wooden bedding (LIGNOCEL^®^ 3–4 S, J. Rettenmaier and Söhne GmbH + Co. KG, Rosenberg, Germany), cotton nestlets (Ancare, UK agents, Lillico, United Kingdom), cocoons (ZOONLAB GmbH, Castrop-Rauxel, Germany), one red plastic house (10 × 9 × 5.5 cm; ZOONLAB GmbH, Castrop-Rauxel, Germany) and tunnels (metal; 12.5 × 5 cm diameter, ZOONLAB GmbH, Castrop-Rauxel, Germany). The animals were kept under standardized laboratory conditions (room temperature 22 ± 2 °C; relative humidity 55 ± 10%) on a light:dark cycle of 12:12 h of artificial light with a 5 min twilight transition phase (lights on from 6:00 a.m. to 6:00 p.m.). Food (ssniff^®^, V1534, Soest, Germany) and tap water were provided *ad libitum*.

### 2.2. Radiometabolism Study

We performed a radiometabolism study to assess the effects of sex and daytime on the route and excretion pattern of TMs. We divided the 32 C57BL/6J experimental mice in two groups containing 16 mice each (8 males and 8 females). At the onset of the study all experimental animals were transferred to sample collection cages equipped with a grid floor (Tecniplast^TM^ Raised Wire Floor). In sample collection cages animals were housed individually and given one day for cage habituation, before intraperitoneally (i.p.) injected with 250 µL of 740 kBq (=20 µCi) ^3^H-testosterone (1,2,6,7-^3^H-testosterone, Amersham Pharmacia Biotech Europe GmbH, Germany) diluted in 1 mL of sterile isotonic saline solution containing 5% ethanol. Group one received the injection at 9:00 a.m. (one hour after the beginning of the light phase; ‘morning group’), whereas the second group received the injection at 9:00 p.m. (one hour after beginning of the dark phase; ‘evening group’). Evening injections were performed under red light. Within the first 24 h we collected feces and urine at 0, 2, 4, 6, 8, 10, 12, 14, 16, 20, and 24 h post injection. Afterwards all excreta were sampled in 12 h intervals until day 5 of the experiment. Collected samples were frozen immediately and stored at −20 °C until analysis.

### 2.3. Diurnal Fluctuations

To assess normal diurnal fluctuations in TM excretion, we collected feces from 16 C57BL/6J (8 males and 8 females) and 8 male B6D2F1 hybrid mice. B6D2F1 hybrids were used as an out-group comparison to assess whether testosterone excretion patterns show strain specific variation. The C57BL/6J mice had previously been used in the radiometabolism experiment and were randomly selected within sexes. Between experiments the re-used mice were provided 8 days to recover where they were individually housed under the conditions described above. At the onset of the study all experimental animals were transferred to sample collection cages, where they were given one day for cage habituation before sample collection started. Sample collection was performed according to the following time intervals starting at 9:00 a.m.: 0, 2, 4, 6, 8, 10, 12, 14, 16, 20, and 24 h.

### 2.4. Human Chorionic Gonadotropin (hCG) Challenge

To physiologically validate the applied EIA we performed a pharmacological stimulation with hCG (Chorulon, Intervet, Vienna, Austria). As hCG mimics the activity of luteinizing hormone (LH), its administration induces testosterone production in Leydig cells and leads to a testosterone surge. The hCG administration was performed in the 8 male C57BL/6J mice, which had been used to assess diurnal fluctuations. The hCG administration was conducted on the following day and we applied a within-subject design to distinguish between the effects of the pharmacological stimulation compared to the naturally occurring circadian rhythms. Each male received 2.5 IU hCG/g body mass injected i.p. at 9:00 a.m. and feces were collected at 0, 2, 4, 6, 8, 10, 12, 14, 16, 20, and 24 h post injection. This dosage has previously been confirmed to induce a testosterone surge [31]. Samples were frozen immediately and stored at −20 °C until analysis.

### 2.5. Castration Experiment

Castration was performed as an additional biological validation for the applied EIA. As castration prevents testicular testosterone production it should reduce male testosterone levels. To confirm the effect of castration on fecal TM levels, fecal samples were collected at three different time points: (a) Two days prior to castration, (b) two days post castration, and (c) two weeks post castration. In total 19 male C57BL/6J mice were castrated at the age of 18 to 42 weeks.

For castration, anesthesia was induced with 4% isoflurane (Isofluran CP^®^, CP-Pharma Handelsgesellschaft mbH, Burgdorf, Germany) in 100% oxygen in an induction chamber and maintained with 1.5–2.5% isoflurane via nose cone. Sterile eye ointment (Artelac^®^ Splash MDO^®^, Bausch and Lomb GmbH, Berlin, Germany) was used to prevent dehydration. Systemic analgesia was provided by meloxicam (1 mg/kg body mass, Metacam 2 mg/mL injection solution for cats, Boehringer Ingelheim Vetmedica GmbH, Ingelheim, Germany), a long-acting non-steroidal anti-inflammatory drug (NSAID) which requires a single dosing a day [32]. Lidocaine/prilocaine was used as topical local anesthetic for the scrotum (Emla Creme, AstraZeneca GmbH, Wedel, Germany). It should be noted here that a single daily dosing with meloxicam could be insufficient to fully control post-op pain in mice [33] and that a subcutaneous injection of lidocaine/bupivacaine at the incision site and/or higher doses of meloxicam (e.g., 5 mg/kg, every 12 h) may improve analgesia post castration [34]. Surgical castration was performed according to Behringer et al. [35]. In short, the scrotum was disinfected and mice were placed in the supine position on a heating pad. Both testicles were pushed down into the scrotal sacs by gently applying pressure to the abdomen. Then, an incision with the length of approximately 1 cm was made through the skin at a right angle to the midline of the scrotal sac. The testes were removed one by one. The membrane covering the testicle was incised and the testicle was carefully pushed out. The vas deferens with the blood vessels running along it was pinched off using two artery forceps. An absorbable suture (3–0) was passed in between the two forceps for ligation. After the forceps were removed, the testicle was dissected from the fat pad and removed. The fat was returned to the scrotal sac and the skin was stitched with a single button suture. After surgery, mice received food pellets that were soaked in water for five days and regular health checks were performed.

### 2.6. Sample Collection

#### 2.6.1. Radiometabolism Study, Diurnal Fluctuations and hCG Challenge

To enable individual sampling and quantitative collection of all voided urine and feces we applied a similar method as described in Touma et al. [16]. Experimental animals were placed individually in type II mouse cages (26.8 × 21.5 × 14 cm, Tecniplast, Buguggiate, Italy) containing a stainless steel wire grid floor (Tecniplast^TM^ Raised Wire Floor, mesh width 7 mm), which was placed in a mouse cage of the same size. All excreta dropped through the bars of the wire floor and were collected in the cage located beneath, which was covered with filter paper to immediately absorb the urine. At each sample point the wire floor cage was placed into a new cage and all excreted fecal pellets were collected for further analyses. To provide time to habituate to the grid floor and the sampling procedure, all animals were transferred to the grid floor cages one day prior to the start of the experiments and samples were collected at regular intervals during this time. To habituate the animals to the single housing condition during the experiments, all mice were separated and placed individually in type IIL mouse cages (36.5 × 20.7 × 14 cm, Tecniplast, Buguggiate, Italy) 10 days prior to the start of the experiment. Cages were equipped with wooden bedding, nesting material, and cardboard tubes as described above. To mitigate potential effects of social isolation and to stimulate reproductive physiology we provided bedding (odor) stimulation of opposite sex conspecifics twice during the habituation period.

#### 2.6.2. Castration Experiment

Since mice had been individually housed for 6 to 32 weeks in type III mouse cages (42.5 × 27.6 × 15.3 cm, Tecniplast, Buguggiate, Italy), they were already habituated to this housing condition. For fecal sample collection mice were transferred to a new home cage, i.e., cages contained wooden bedding, nest material and standard enrichment as described above. Soiled bedding material without feces was scattered on the new bedding to minimize distress accompanied with the cage change. After a period of 24 h, all dry fecal pellets were collected using forceps. Pellets contaminated with urine were excluded. Collected samples were frozen immediately and stored at −20 °C until further analyses were performed [30,36].

### 2.7. Sample Analysis

To determine the route and time course of testosterone excretion, we processed the samples collected in the radiometabolism experiment: Urine spots were cut out from the filter paper and slit into 2–3 cm^2^ pieces. Fecal samples were homogenized with a mortar and pestle before extracted according to the protocol described in Touma et al. [16]. After extraction, the radioactivity of each sample was determined with a liquid scintillation counter (Tri-Carb 2100TR, Packard Instruments, Meriden, CT, USA). Delay times were assessed based on peak excretion patterns and the ratio of recovered radioactivity in urine and feces provided information about the excretion route. The total recovery rate of the radiolabeled metabolites was calculated as the sum of recovered radioactivity in urine and feces divided by the total amount of administered radioactivity.

To characterize the type and relative abundance of ^3^H-testosterone metabolites we performed a reversed-phase HPLC. Only feces containing peak concentrations of radioactivity were used and the analyses were conducted according to the methods described in Touma et al. [16]. Briefly, a Novopack C18 column (3.9 × 150 mm, WAT086344, Waters) and a linear water/methanol gradient (20–100%, flow rate: 1 mL/min, three fractions per min) was used for separation of the metabolites. The immunoreactivity of the HPLC fractions was measured with two different enzyme immunoassays (EIAs): A testosterone EIA and an epiandrosterone EIA. Details of both EIAs including cross-reactions are given in Palme and Möstl [37]. The better performing testosterone EIA was then selected to determine the amount of TM in feces collected to assess diurnal fluctuations, as well as after the hCG administration and the castration. Sample extraction was performed as described above and according to the protocol given in Palme et al. [38]. All assays were conducted blinded and samples were run in duplicates to receive results.

The testosterone EIA utilizes an antibody produced in rabbits against testerone-3-CMO:BSA (working dilution 1:20,000) and 5α-androstane-3β,17β-diol-3-HS-DADOO-biotin as label (dilution 1:160,000). Further details of the EIA procedure are given in Palme and Möstl [37]. The sensitivity of the whole analysis was 0.21 ng/0.05 g feces. Intra- and inter-assays coefficients of variation of high/low concentration pool samples were 2.1/2.9% and 6.7/7.2%, respectively.

### 2.8. Statistical Analysis

Statistical analyses were performed with IBM SPSS Statistics 24 and R 3.3.2 and figures were created in Sigma Plot 13.0.

#### 2.8.1. Radiometabolism Study

To test if the recovery rate of the radiolabeled testosterone differed between the sexes or the experimental treatment groups we applied a Mann–Whitney U test. We applied a paired T-Test to compare the amount of excreted feces between day and night times. To determine which factors influenced the excretion pattern in fecal or urinary samples we ran general linear mixed effects models (LMM) with the amount of excreted testosterone (per 50 mg feces) or urine (total amount of collected urine for the given collection period) as the dependent variable and time point of sample collection, sex and experimental treatment group (morning or evening injection) as fixed factors. Animal ID was included as random factor to control for non-independence of samples from the same individual. To assess which factors influenced the delay times to peak excretion in fecal and urinary samples, we ran linear models (LM) with the delay times to peak excretion as the dependent variable and sex and treatment group as fixed factors.

#### 2.8.2. Diurnal Fluctuations

To test whether sex influenced TM levels in C57BL/6J mice, we ran a LMM with testosterone levels (ng/50 mg feces) as dependent variable, sex as fixed factor and animal ID as random factor. We assessed the effect of daytime on TM levels separately for male and female C57BL/6J mice in performing LMMs with TM levels as dependent, time point of sample collection as fixed and animal ID as random factor. To assess whether male fluctuations in TM levels were similar across genetic backgrounds we performed a Kendall W test.

#### 2.8.3. hCG Challenge and Castration Experiment

To test if hCG administration increased TM levels, we compared individual peak levels before hCG administration with respective TM levels at the same daytime after hCG administration in a Wilcoxon Signed Rank test. Finally, to assess whether castration reduced male TM levels, we performed a One Way Repeated Measures Analysis of Variance on ranks.

### 2.9. Animal Welfare

The radiometabolism study, the diurnal fluctuations study and the hCG challenge experiment has been discussed by the Institutional Ethics Committee of the University of Veterinary Medicine Vienna in accordance with Good Scientific Practice guidelines and has been approved by the Austrian Federal Ministry for Science and Research (Reference number: BMWFW-68.205/0056- WF/V/3b/2017).

The castration study was performed according to the German Animal Welfare Act and the Directive 2010/63/EU for the protection of animals used for scientific purposes. Animal housing and husbandry were approved by the Berlin State Authority (“Landesamt für Gesundheit und Soziales”, permit number: G 0053/15). Mice were previously used in an experiment classified as mild with regards to animal suffering (as defined by current EU regulations on severity classification of animal studies). After castration and re-socialization in groups of three to four, the mice were rehomed.

## 3. Results

### 3.1. Radiometabolism Study

#### 3.1.1. Time Course and Route of ³H-testosterone Excretion

The mean total recovery of the administered radioactivity was 57 ± 8% in feces and urine samples combined over all animals. No difference in the recovery rate was detected between the experimental treatment groups (morning injection: 56.6 ± 7.5%, evening injection: 57.1 ± 4.8%; Mann–Whitney U test: U = 125.5, N = 32, *p* = 0.94) or between sexes (males: 56.4 ± 6.3%, females: 57.3 ± 6.3% Mann–Whitney U test: U = 145.5, N = 32, *p* = 0.52). Interestingly though, we found that males secreted significantly more radiolabeled TMs via the feces than females (males: 59.0 ± 7.3%, females: 49.5 ± 5.5%; Mann–Whitney U test: U = 39, N = 32, *p* < 0.001), whereas females showed higher proportions of radioactivity in the urine compared to males (males: 41.0 ± 3.7%, females: 50.3 ± 5.5%; *p* < 0.001).

**Figure 1:**
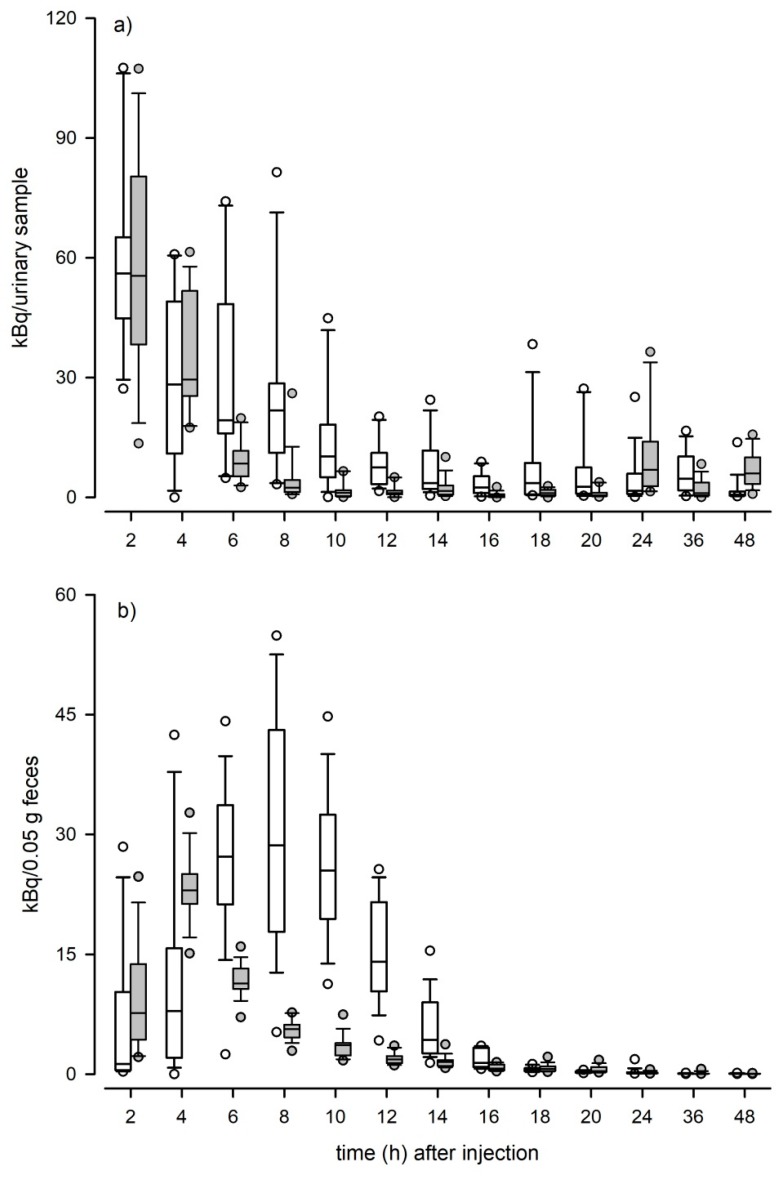
Time course (first 48 h) of ^3^H-testosterone excretion in (**a**) urinary and (**b**) fecal samples of male and female C57BL/6J mice. White boxplots (n = 16) represent data obtained from mice that received the ^3^H-testosterone in the morning (one hour after the onset of the light phase) and grey boxplots (n = 16) represent data from mice that received the ^3^H-testosterone in the evening (one hour after the onset of the dark phase). Dots are outliers with values between 1.5‒3.0× interquartile range.

Overall, animal defecation rates were significantly dependent on daytime (Paired *t*-Test: *t* = −35.95, *p* < 0.001): Experimental mice produced on average 8.02 g feces during the 12 h collection periods from 9 p.m. to 9 a.m. compared to 3.22 g feces during the 12 h collection periods from 9 a.m. to 9 p.m. The excretion of radiolabeled TMs varied significantly over time and showed different patterns in urinary and fecal samples (LMM feces: F_15, 464_ = 39.33, *p* < 0.001; LMM urine: F_15, 464_ = 57.65, *p* < 0.001). Within both, urinary and fecal samples, we found a significant effect of the time of injection on the excretion pattern (LMM feces: F_1, 30_ = 48.21, *p* < 0.001; LMM urine: F_1, 30_ = 19.07, *p* < 0.001): In fecal samples, animals that received the radiolabeled testosterone in the evening showed a faster rise in excretion rates and excreted the radiolabeled testosterone more quickly compared to animals that received the radiolabeled testosterone in the morning (Figure 1b). In urinary samples, animals from both treatment groups showed an immediate rise in excretion rates, but animals from the evening group excreted the radiolabeled testosterone faster compared to animals from the morning group (Figure 1a). Within both sample types no sex-specific differences in the excretion patterns were detected (LMM feces: F_1, 29_ = 1.64, *p* = 0.21; LMM urine: F_1, 29_ = 1.55, *p* = 0.22) and the vast majority of radiolabeled metabolites in feces (97.3 ± 1.5 %) and urine (84.6 ± 12.2%) was excreted within the first 24 h.

**Figure 2:**
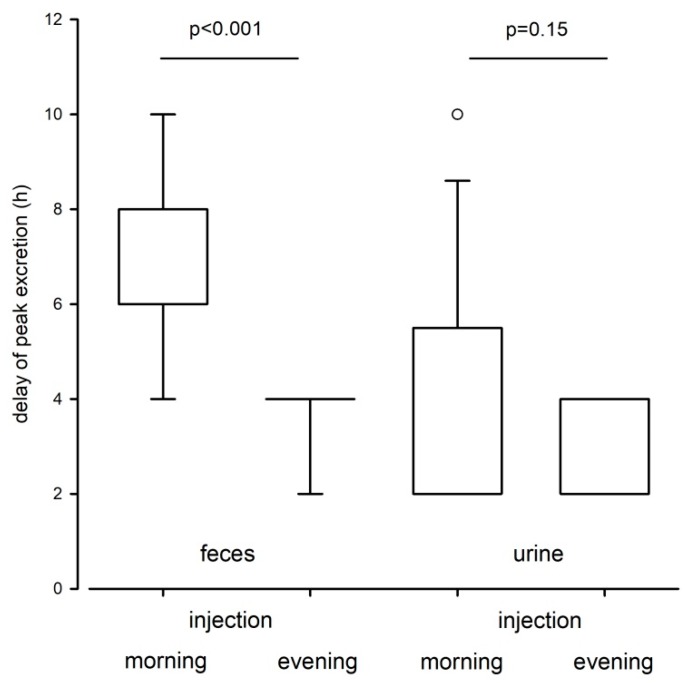
Delay time in hours to peak excretion in urinary and fecal samples of C57BL/6J mice. Peak excretion times are depicted separately for the morning (n = 16) and evening (n = 16) injection group. Mice from the morning group received the ^3^H-testosterone one hour after the onset of the light phase, whereas mice from the evening group received the ³H-testosterone one hour after the onset of the dark phase. Dots are outliers with values between 1.5‒3.0× interquartile range.

We further determined the delay time to peak excretion in urinary and fecal samples. In urine, peak radioactivity was recovered in the first samples collected after the administration of ^3^H-testosterone, i.e., after 2 h (range 2–8). No differences were found between sexes (LM: F_1, 29_ = 1.22, *p* = 0.28), or between animals from the morning or evening injection group (LM: F_1, 29_ = 2.17, *p* = 0.15; Figure 2). In feces, peak excretion times were significantly affected by the time of injection (LM: F_1, 30_ = 51.91, *p* < 0.001; Figure 2): In the evening group peak radioactivity was recovered after 4 h (no range) and in the morning group after 8 h (range 4–10). Again, no sex-specific differences were discovered in fecal peak excretion times (LM: F_1, 29_ = 0.06, *p* = 0.81).

#### 3.1.2. Characterization of Fecal ^3^H-testosterone Metabolites

**Figure 3:**
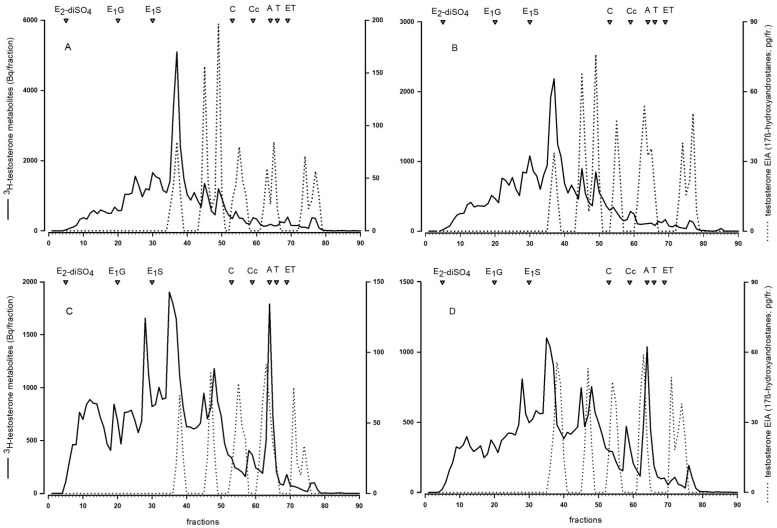
Reversed-phase HPLC separation of fecal ^3^H-testosterone metabolites of male (**A**,**B**) and female (**C**,**D**) mice from the morning (**A**,**C**) and evening (**B**,**D**) injection group. Radioactivity (solid line) of each fraction was determined by liquid scintillation counting. Immunoreactivity (dashed line) was tested with the testosterone enzyme immunoassays. Open triangles mark the approximate elution positions of respective standards (E2-diSO4 = 17β-estradiol-disulfate, E1-G = estrone-glucuronide, E1S = estrone-sulfate, C = cortisol, Cc = corticosterone, A = androstenedione, T = testosterone, ET = epitestosterone).

The injected ^3^H-testosterone was heavily metabolized and HPLC separations revealed several peaks of radioactivity, indicating that a large number of metabolites were formed. We found clear differences in the excreted metabolites between males and females, but the pattern was identical between animals that received the radiolabeled testosterone in the morning or evening (Figure 3). Females showed a broader spectrum of metabolites of different polarity compared to males and also showed a pronounced peak eluting around androstenedione, which was absent in males. Several ³H-metabolites also reacted with the testosterone EIA.

### 3.2. Evaluation of Diurnal Fluctuations

**Figure 4:**
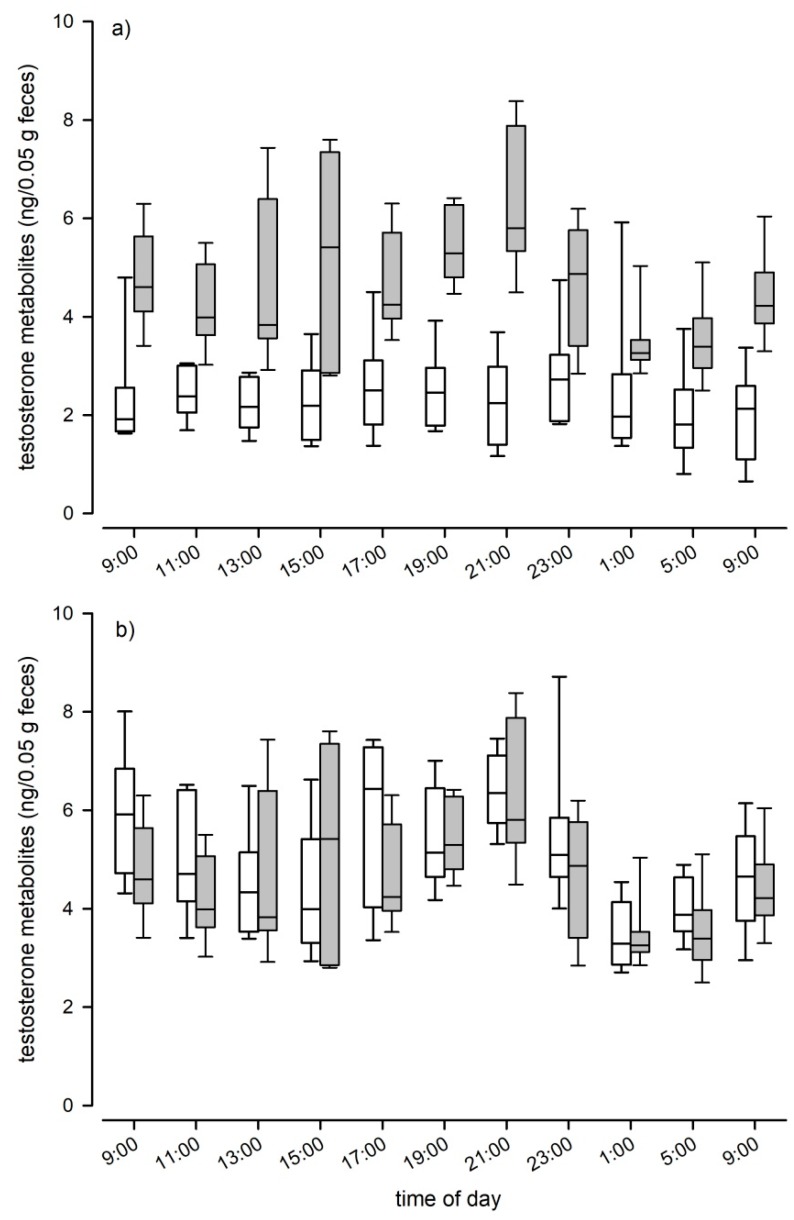
Testosterone metabolite levels over the course of 24 h in (**a**) male (grey boxplots; n = 8) and female (white boxplots; n = 8) C57BL/6J mice and (**b**) male C57BL/6J (grey boxplots; n = 8) and male B6D2F1 hybrids (white boxplots).

When we assessed fecal TM levels of C57BL/6J mice, we found that testosterone levels differed significantly between sexes over the course of a day (LMM: F_1, 14_ = 49.87, *p* < 0.001; Figure 4a). Males had twice the concentration of females, as males showed an average testosterone level of 4.65 ng/50 mg feces (± 1.34 SD) compared to 2.35 (± 0.9 SD) in females. Males also showed significant variation in TM levels over the course of the day (LMM: F_10, 65_ = 4.74, *p* < 0.001; Figure 4a), whereas female TM levels did not show such fluctuations (LMM: F_10, 69_ = 1.17, *p* = 0.324; Figure 4a). We found no evidence that genetic background affected TM levels, as absolute levels and the pattern of episodic fluctuations were comparable between C57BL/6J and B6D2F1 hybrid males (Kendall W test: W = 0.38, df = 10, *p* < 0.001; Figure 4b). B6D2F1 hybrid males had an average testosterone level of 4.92 ng/50 mg feces (± 1.44 SD). Both strains showed two peaks in their testosterone levels, the first one occurred between 3 p.m. and 5 p.m. and the second and most pronounced peak was reached at approximately 9 p.m., one hour after the onset of the dark phase. Nadir levels of testosterone were observed in fecal samples that were collected around midnight (see Figure 4b).

### 3.3. Physiological and Biological Validation

**Figure 5:**
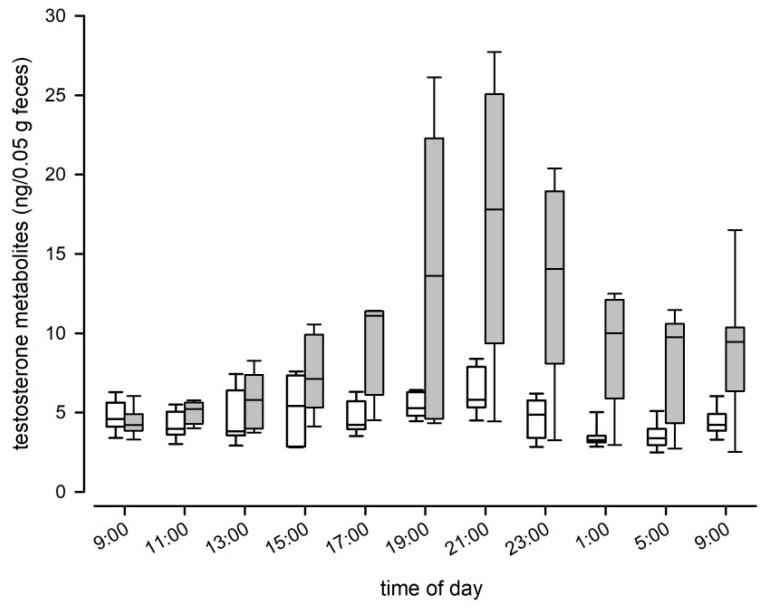
Testosterone metabolite levels in male C57BL/6J mice (n = 8) for 24 h without prior hCG administration (baseline; white boxplots) and for 24 h post hCG administration (grey boxplots).

**Figure 6:**
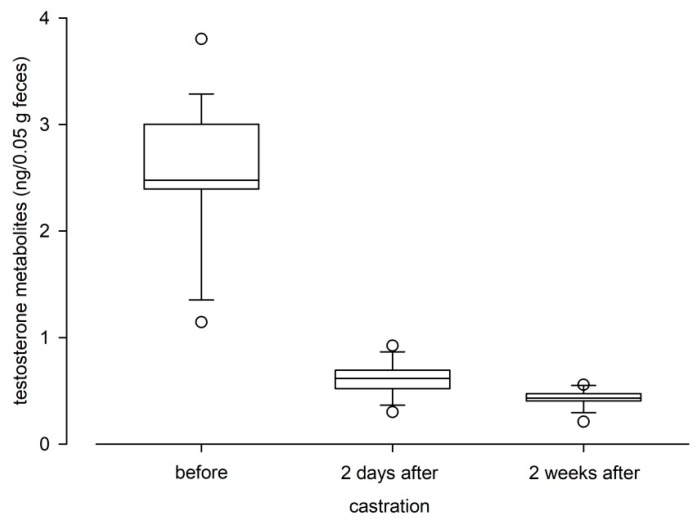
Testosterone metabolite levels in male C57BL/6J mice before, two days and two weeks after castration. Dots are outliers with values between 1.5‒3.0× interquartile range.

The hCG administration significantly increased TM levels in experimental male mice (Wilcoxon Signed Rank test: Z = −2.1, *p* = 0.036). TM levels increased from a median of 4.36 ng/50 mg feces during the 24-h observation period before administration to 8.78 ng/50 mg feces post administration, and a clear peak was observed 12 h after the hCG injection (Figure 5). Castration significantly reduced testosterone metabolite levels in males (RM Anova on ranks: Χ^2^ = 34.4, *p* < 0.001; Figure 6). Castration had an immediate and strong effect on male TM levels, as levels dropped below levels found in females within two days.

## 4. Discussion

Our multiphase validation experiment for the non-invasive measurement of TMs in fecal and urinary samples of mice provided several interesting results and we start our discussion in highlighting the sex-specific differences we discovered. First, we found a significant sex difference in the route of TM excretion. Males excreted 59% of the radiolabeled testosterone via the feces. This rate was about 10% higher compared to females, which in turn, excreted a bigger proportion of radiolabeled testosterone via the urine. A previous study in laboratory mice has also reported sex-specific differences in the excretion route of corticosterone metabolites (CMs), where males excreted higher proportions of radiolabeled CMs via the feces compared to females [16]. This difference was even more pronounced, as males excreted about 73% of radioactive corticosterone via the feces, whereas females excreted only about 53%. These results show that sex-specific differences in hormone metabolite excretions are common in mice and that males on average might excrete a bigger proportion of hormone metabolites via feces compared to females. The reason for this difference is unclear. Similar studies investigating TMs excretion in red squirrels [18] and bank voles [21] did not find differences between males and females in the route of excretion. Interestingly though, the study in bank voles found sex-specific differences in the excretion of CMs, as males excreted significantly more radiolabeled corticosterone via feces than females [21], highlighting the importance to test for sex-specific differences in excretion routes in each species.

Secondly, we also found that males and females differed in the formed fecal TMs. Female reversed-phase HPLC immunograms showed a more diverse pattern, they contained more and different peaks compared to male immunograms, indicating that females form a broader range of different TMs compared to males. Similar to our finding in mice, the type of TMs excreted in feces of red squirrels also showed sex-specific differences [18,19]. Females showed three peaks of immunoreactive TMs in immunograms, whereas males only showed two. Also in bank voles, testosterone was heavily metabolized with clear sex-specific differences [21], suggesting that sex-specific differences in the formed fecal TMs might be common in rodents. In summary, these results urge caution in comparing fecal TMs between males and females but do not diminish the applicability of the EIA to measure fecal TM in male and female mice. The reversed-phase HPLC in our study also showed that plasma testosterone is heavily metabolized and that there is hardly any testosterone left in feces, only its metabolites. This result emphasizes the use of group specific EIAs designed to specifically detect hormone metabolites rather than their actual plasma hormones in fecal samples [12,14].

Overall, we found much variation in the excretion pattern of radiolabeled TMs in urinary and fecal samples but within the excretion routes the time course of radiolabeled testosterone excretion did not differ between sexes. The vast majority of radiolabeled metabolites were excreted within the first 24 h and day time of the injection significantly affected the excretion patterns. In both fecal and urinary samples, animals that received the ³H-testosterone in the evening excreted the radiolabeled testosterone faster compared to animals that received the ^3^H-testosterone in the morning, indicating a faster excretion when animals are more active, i.e., during the dark phase for nocturnal animals. This may be mainly related to the higher (in this study on average 2.5 times) defecation rates during this phase. Most steroids are quickly removed from the circulation and are heavily metabolized by the liver and excreted via the kidneys into the urine or via the bile into the duodenum. The delay time between the secretion of a hormone and the excretion of its metabolites is dependent on the individuals’ gut passage time. Depending on the species, this time interval can be less than 30 min or more than a day [13]. In mice, the gut passage time is estimated to be approximately 9–10 h [39,40] and our data clearly show that even within a species this time interval can vary strongly depending on the circadian rhythm. Knowing the intestinal gut passage time of a species is highly important when examining the endocrine status of animals based on excreta [13] and studies have to consider animal activity patterns in order to properly assess TM levels.

We also found that the elimination of radioactivity via urine occurs faster than via feces. Urinary excretion peaks were already observed after 2 h. This finding is in line with other studies that investigated the excretion pattern of radiolabeled CM in mice and rats, which discovered that excretion peaks are usually found within the first few hours of sample collection [16,41]. In comparison, excretion peaks in feces were observed later and either occurred after 4 h when the samples were collected during the dark phase, i.e., when animals were more active, or after 8 h when samples were collected during light phase, i.e., when animals were in their resting period. Mice show phases of activity scattered all around the day; however, they are nocturnal and their main activity phase is during the dark phase. When animals are more active, more feces can be collected. This could explain the earlier detection of radiolabeled TMs in those samples collected during the dark phase. Importantly, fluctuations in defecation rate can result in distortions of fecal hormone metabolite concentrations without any changes in bioactive hormone levels.

Recently it has been demonstrated that different steroids can show different time courses in their excretion. For example, in bank voles corticosterone was excreted faster than testosterone [21]. In urinary samples excretion peaks were observed after 4 h for corticosterone and after 6 h for testosterone. In fecal samples, excretion peaks occurred after 6 and 8 h, respectively. In mice, if we compare our results with those reported in Touma et al. [16], no difference in the time course of corticosterone and testosterone excretion was observed in urine and solely a small difference occurred in feces: Only when animals were inactive, peak excretion differed between steroids. Surprisingly however, we showed that testosterone was excreted faster compared to corticosterone, i.e., after 8 versus 10 h. It is unclear what could have postponed the metabolism of corticosterone over testosterone but given that the animals were kept under similar conditions one potential explanation for the slightly delayed excretion of corticosterone could be plasma binding proteins. Plasma binding proteins lead to a slower removal of steroids from the circulation and are known modulators of steroid action in mammals [42]. Alternatively and not mutually exclusive, this difference in peak excretion time between corticosterone and testosterone could also be explained by an enterohepatic recirculation of some metabolites [13].

Our experiment clearly showed that testosterone metabolism in mice is not affected by the time of injection, i.e., depending on the activity pattern of the animals, as there were no differences in the metabolite patterns between injection groups in the reversed-phase HPLC immunograms. Thus, the delay time between injection and excretion via feces had no effect on the type of formed TMs. We could also show that the chosen testosterone EIA (measuring 17β-hydroxyandrostane), first described by Palme and Möstl [37], is suitable for reliably measuring fecal TM in mice. The EIA was able to detect several metabolites in the radiometabolism study and the hCG challenge experiment confirmed the physiological sensitivity of the selected EIA as we detected a clear and significant increase in the fecal TM concentrations after hCG administration. Peak values were detected approximately 12 h after injection. Thus, peak excretion occurred 4 h later compared to the radiometabolism study. This delay time can most likely be explained by the time required for the hypothalamic–pituitary–gonadal axis to induce testosterone secretion. We could further confirm the biological validity of the applied EIA, in measuring an immediate and steep decline in TM levels after castration. We are confident that our applied EIA can be used to reliably assess male and female TM levels, even though the physiological and biological validations were performed solely in males. Validation of the assay in females is not straight forward, as females do not carry a grand scale testosterone producing organ like males, that could be removed or pharmacologically stimulated to trigger a significant alteration in testosterone production. However, to test the sensitivity of the selected EIA for TM levels in females and to further test its sensitivity for TM overall, we compared the natural TM levels of males with those of non-pregnant females. The EIA was able to detect the low concentrations found in females and showed the expected differences between the sexes: Males had on average double the TM levels than females and showed clear, episodic fluctuations in TM levels over the course of a day. The highest TM concentrations were found between 3 p.m. and 5 p.m. and at 9 p.m. (shortly after the onset of the dark phase). Considering the intestinal gut passage time, testosterone peaks in males likely occur early in the morning around 7 a.m., which is at the end of the dark phase when they are most active and around noon, in the middle of the light phase. Similarly to our result, in bank voles the highest testosterone production also seems to occur around morning and before noon [21]. The daily fluctuations in TM concentrations were highly similar in male C57BL/6J and B6D2F1 hybrid males, indicating that male genetic diversity had no effect on TM levels and that there are no strain specific differences in TM excretion patterns in mice.

## 5. Conclusions

Analyzing hormone metabolites in excreta appears to be a quick and an easy solution for many problems. Unfortunately, this is not true and there is a strong need to carefully validate assays analytically, physiologically, and biologically in every new species as the metabolism and excretion of hormone metabolites differ significantly between species and sometimes even between sexes [10]. Our multi-phase validation experiment identified and confirmed the testosterone EIA to be suitable for measuring TM in male and female mice. Furthermore, we were able to extend our knowledge on the endocrine physiology of mice. Our non-invasive method to assess fecal TMs can be widely used to investigate hormone–behavior relationships, and it can be applied in various other research areas such as reproduction, animal welfare, ecology, conservation and biomedicine.
